# The global diet quality score as an indicator of adequate nutrient intake and dietary quality – a nation-wide representative study

**DOI:** 10.1186/s12937-024-00949-x

**Published:** 2024-04-17

**Authors:** Marina M. Norde, Sabri Bromage, Dirce M. L. Marchioni, Ana Carolina Vasques, Megan Deitchler, Joanne Arsenaut, Aline M. de Carvalho, Lício Velloso, Walter Willett, Edward Giovannucci, Bruno Geloneze

**Affiliations:** 1 Obesity and Comorbidities Research Center, University of Campinas, Campinas, SP Brazil; 2 Institute of Nutrition, Mahidol University, Phuttamonton, Thailand; 3 Department of Nutrition, Harvard T. H. Chan School of Public Health, Boston, MA USA; 4 Department of Nutrition, School of Public Health of the University of Sao Paulo, Sao Paulo, SP Brazil; 5 School of Applied Sciences, University of Campinas, Limeira, SP Brazil; 6 Intake-Center for Dietary Assessment, FHI 360, Washington, DC USA; 7 Department of Epidemiology, Harvard T. H. Chan School of Public Health, Boston, MA USA

**Keywords:** Dietary risk, Dietary quality metrics, Sustainable developmental goal, Nutrient adequacy, Double burden of malnutrition, Brazil, Dietary diversity, Ultra-processed food

## Abstract

**Background:**

The Global Diet Quality Score (GDQS) was developed to be a simple, timely and cost-effective tool to track, simultaneously, nutritional deficiency and non-communicable disease risks from diet in diverse settings. The objective was to investigate the performance of GDQS as an indicator of adequate nutrient intake and dietary quality in a national-representative sample of the Brazilian population.

**Methods:**

Nationally-representative data from 44,744 men and non-pregnant and non-lactating women aging ≥ 10 years, from the Brazilian National Dietary Survey were used. Dietary data were collected through two 24-h recalls (24HR). The GDQS was calculated and compared to a proxy indicator of nutrient adequate intake (the Minimum Dietary Diversity for Women—MDD-W) and to an indicator of high-risk diet for non-communicable diseases (caloric contribution from ultra-processed foods—UPF). To estimate the odds for overall nutrient inadequacy across MDD-W and GDQS quintiles, a multiple logistic regression was applied, and the two metrics’ performances were compared using Wald’s post-test.

**Results:**

The mean GDQS for Brazilians was 14.5 (0–49 possible range), and only 1% of the population had a low-risk diet (GDQS ≥ 23). The GDQS mean was higher in women, elderly individuals and in higher-income households. An inverse correlation was found between the GDQS and UPF (rho (95% CI) = -0.20(-0.21;-0.19)). The odds for nutrient inadequacy were lower as quintiles of GDQS and MDD-W were higher (p-trend < 0.001), and MDD-W had a slightly better performance than GDQS (p-diff < 0.001). Having a low-risk GDQS (≥ 23) lowered the odds for nutrient inadequacy by 74% (95% CI:63%-81%).

**Conclusion:**

The GDQS is a good indicator of overall nutrient adequacy, and correlates well with UPF in a nationally representative sample of Brazil. Future studies must investigate the relationship between the GDQS and clinical endpoints, strengthening the recommendation to use this metric to surveillance dietary risks.

**Supplementary Information:**

The online version contains supplementary material available at 10.1186/s12937-024-00949-x.

## Background

The world, especially in low- and middle-income countries, is witnessing a global syndemic of obesity, undernutrition, and climate change [1]. Dietary factors contribute substantially to the global syndemic as the rise of monocultures and livestock in food production are major factors contributing to a progressive loss in diet biodiversity, and increased consumption of low-nutrient and high-energy dense foods, with outstanding impacts on both, the environment and public health [2]. The unequal incentives between high and low-middle income countries to food commodities production and trading, to the detriment of smaller stakeholders contributing to local food production and supply, further disproportionately affect food sovereignty and food security across nations [3]. According to the Global Burden of Disease 2019 estimations, diet risk factors along with malnutrition are the most important behavior risk factor for all-cause mortality worldwide, accounting for an estimated 145 deaths annually per 100,000 inhabitants [4].

Given the importance of diet for health and living conditions, international organizations, such as the United Nations, are seeking indicators to monitor dietary risks across countries [5]. Many United Nations Sustainable Development Goals (UN-SDG) are, in many ways, permeated by diet quality, with emphasis to UN-SDG number 2, which stands for eradicating all forms of malnutrition through sustainable food systems promotion [6]. However, a harmonized indicator for diet quality to be tracked globally, capturing diet-related risk for both sides of malnutrition, across different cultures and resource scenarios is still lacking, which poses a serious limitation for UN-SDG surveillance [7].

The Global Diet Quality Score (GDQS) was developed to be a food-based, simple, timely and cost-effective tool to track, simultaneously, nutritional deficiency and non-communicable disease (NCD) risks from diet, aiming to be applied in a diverse set of food cultures, allowing comparison of dietary quality across nations and over time [8]. Among the advantages of the GDQS are its food-based format, which eliminates the need for food composition tables information, and the fact that it can be used in both secondary data and in primary data collection, for which a time saving smart-device app, suitable for low-resources settings, was developed to support dietary assessment [9].

The GDQS predictive validity has already been shown in studies conducted in Mexico [10, 11], India [12, 13], Thailand [14], China [15], Ethiopia [16, 17], Sub-Saharan African countries [18], and the United States of America [19, 20]. In those studies, the validation was conducted comparing the GDQS performance in predicting nutrient inadequacy, weight gain, and worse metabolic biomarkers profile, with that of two well-known and widely used diet metrics: the Minimum Dietary Diversity score for Women (MDD-W), well-established as a good proxy for the probability of adequate micronutrient intake in women [21]; and the Alternative Healthy Eating Index (AHEI), an indicator of diet-related NCD-risk [22]. In general, the GDQS displayed similar performance to the MDD-W for assessing probability of micronutrient adequacy and to the AHEI for assessing risk for NCD-related outcomes, with the advantage that it is a single metric to help surveillance under- and overnutrition risk from diet simultaneously and, different than AHEI, is independent of food composition tables availability [8].

However, there are some gaps that must be addressed for the GDQS to claim its worldwide applicability, such as the fact that it still lacks validation for some regions of the World, such as countries located South from the equator line [8]. In this context, Brazil emerges as an interesting country to undergo evaluation; it is the largest South American country, with a continental territory, which makes it a promising geographic scenario for food culture diversity to be explored [23].

As in most developing countries, Brazil deals with a co-existence of malnutrition in all its forms, and their impacts on population health [24, 25]. Since 1975, the prevalence of obesity in Brazil has increased progressively each year, particularly in socio-economically vulnerable strata [26, 27], while, since the 2015 economic crisis, food insecurity in Brazil has increased from 22.6% in 2013 to 36.7% in 2018 and became even worse during COVID-19 pandemic, reaching 58.7% during the first semester of 2022 [28, 29].

Therefore, given the promising applicability of the GDQS to surveillance dietary risks for all forms of malnutrition, and considering the strategic geopolitical position of Brazil alongside the critical scenario for food insecurity established in the last decades in the country, the present study aimed at investigating the applicability of GDQS as a food-based dietary metric for dietary quality in a nationally representative sample of the Brazilian population, comparing its performance with that of MDD-W to predict inadequate nutrient intake, considering possible differences in performance across sex, age ranges, and Brazilian regions.

Furthermore, to offer insights on GDQS potential as NCD-risk indicator, the GDQS correlation with energy intake from ultra-processed foods (UPF), an food-based indicator of unhealthy dietary habits, higher dietary risk for obesity and its comorbidities, and low adherence to the Brazilian Food Guide recommendations, was investigated.

## Methods

Secondary data analysis was conducted with the publicly available and nationally representative dataset the Household Budget Survey (HBS) of Brazil, for which data collection took place between 2017 and 2018. The Brazilian HBS is coordinated by the Brazilian Institute of Geography and Statistics on a ten-year interval, and its main purpose is to survey household budget structures (e.g., products acquisitions, services requirements, and income), nutritional status, and living conditions of families in Brazil [30].

As part of 2017–2018 HBS, a subsample of 46,164 individuals 10-years or older, living in one of the 20,112 randomly selected households (out of the 57,920 households from the original sample), were invited to answer two non-consecutive days 24-h recalls (24HR), carried out by trained research agents in the household, as well as information on current supplement use, and diet modification. The individual dietary assessment phase of the HBS, also called the Brazilian National Dietary Survey (BNDS), preserves the nationwide representativeness of the original sample, respecting the two-stage cluster sampling (census sectors and households), and geographical and sociodemographic stratifications, based on the 2010 Demographic Census [30].

For the present study, all individuals that participated in the BNDS 2017–2018 were included in the analysis. Pregnant and lactating women were excluded, and all the analyses were stratified for self-declared sex (men/women), age ranges (adolescents, 10 to 19 years/adults, 20 to 59 years/elderly, ≥ 60 years), and geographical regions (North/Northeast/Southeast/South/Midwest Brazil, Supplemental Figure S 1).

Information on urban or rural area of residency, sex, age, self-declared skin color, educational status, and household per capita income were collected at the first visit interview and were used as covariates in the present study analysis.

The BDNS publicly available data does not identify participants or households other than rural/urban locality and the geographical code of the Brazilian State. Therefore, the usage of secondary data by the present study waives approval from an Ethics Committee and is in accordance to the Helsinki Declaration and the Ethical Guidelines from the Council for International Organizations of Medical Sciences.

### Dietary assessment

In the BNDS assessment, dietary data were collected through two non-consecutive days 24HR, carried out by trained research agents, following the Automated Multiple Pass Method [31], guided by a tablet app developed specifically for the structured interview [30, 32]. Most of the sample answered both 24HR (*n* = 38,854; 99.9%) and those who did not answer the second measurement were kept in the dataset with a single 24HR information.

Participants were asked to list all the foods and beverages consumed the day before the interview without interruptions from the research agents. After that, the participants were asked about further details on culinary techniques, amount, added items (e.g.: olive oil, butter, ketchup, sugar, salt, sweeteners, honey, sauces, grated cheese, milk cream), and occasion and place when and where each food item was consumed. At the end of the interview, participants were also inquired about the usage of nutritional supplements and the existence of any special condition that could restrict their dietary intake (i.e.: actively trying to lose weight; high blood pressure, high cholesterol, diabetes, or heart disease treatment; and others) [30]. The nutrient content of foods was determined using the Brazilian Food Composition Table (TBCA-USP), version 7.0 [33].

### Scoring diet metrics

To conduct the analysis, five diet metrics were calculated using the first 24HR data: (1) GDQS; its two sub-metrics, (2) GDQS + and (3) GDQS -; (4) the MDD-W; and (5) the percentage of dietary caloric contribution from UPF.

To score all five dietary metrics, 24HR mixed dishes were disaggregated into ingredients using standard recipes for the Brazilian cuisine, and yield and nutrient retention factors were applied from standard references [34–38], further explained elsewhere [39]. Food classifications were double-checked by two researchers.

The GDQS scores the dietary daily intake of 25 food groups, in grams, according to their contribution to increase or decrease the overall quality of individual diets (ranging from 0 to 49). The 25 food groups of GDQS can be separated into the so-called “healthy foods” – comprising 16 food groups which intake increase the overall diet quality score (dark-green leafy vegetables, deep-orange vegetables, deep-orange fruits, deep-orange tubers, cruciferous vegetables, other vegetables, citrus fruits, other fruits, fish and shellfish, poultry and game meat, legumes, nuts and seeds, low-fat dairy, eggs, whole grains, and liquid oils); the “unhealthy foods” – comprising seven food groups which intake decrease the overall diet quality score (white roots and tubers, processed meat, refined grains and baked goods, sugar-sweetened beverages, juice, sweets and ice creams, and purchased deep-fried foods); and two food groups classified as “unhealthy in excessive amounts”, which optimal intake increases while excessive intake decreases the overall diet quality score (red meat, and high-fat dairy). The “healthy” and “unhealthy” food groups can be scored separately into sub metrics GDQS + (ranging from 0 to 32) and GDQS- (unhealthy food groups, including those claimed unhealthy in excessive amounts, ranging from 0 to 17), respectively. Further details on GDQS scoring methods can be found elsewhere [8].

MDD-W, on its turn, scores 1 point for the intake of each one of the 10 predefined food groups: grains, white roots, and tubers and plantains; pulses; nuts and seeds; milk and milk products; meat poultry, and fish; eggs; dark-green leafy vegetables; other vitamin A rich fruits and vegetables; other vegetables; other fruits. Those food groups were defined based on their importance for diet diversity and, consequently, adequacy of micronutrient intake (vitamin A, thiamine, riboflavin, niacin, vitamin B6, folate, vitamin B12, vitamin C, calcium, iron, and zinc), especially for women. MDD-W is usually applied in a dichotomous way, in which minimum diet diversity is achieved when at least 5 out of 10 food groups are included in individuals’ diets. For the present study, MDD-W was scored from 0 to 10, adding one point every time individual diets had one or more food items consumed in more than 15 g/day from each of the 10 predefined food groups [21].

Even though MDD-W has its main usage directed to women, it can be used as a proxy of micronutrient adequacy in other groups [21]. For this reason, MDD-W was also investigated in men in this study [21].

The GDQS performance in predicting overall probability of nutrient adequate intake was compared to that of MDD-W because the former is a well-established proxy for the probability of adequate micronutrient intake [21].

Given the importance of UPF as an indicator of unhealthy dietary habits [40], higher dietary risk for obesity and its comorbidities [41], and low adherence to the Brazilian Food Guide recommendations [42], we investigated its correlation with the GDQS. UPF compose one of the four food groups determined by the NOVA classification system [40]. For this study, foods were manually classified as pertaining to UPF category according to the NOVA classification system described in detail elsewhere [40], and the calorie from UPF was divided by the total caloric intake of the diet to generate a percentage of caloric contribution from UPF for the first day of dietary data collected.

### Nutrient intake

Predicted individual usual intakes of protein, total fat, saturated fatty acids (SFA), monounsaturated fatty acids (MUFA), polyunsaturated fatty acids (PUFA), fiber, calcium, iron, zinc, vitamin A, folate, and vitamin B12 intakes, from two 24HR, as well as energy, were estimated using the National Cancer Institute (NCI) method to adjust for within-person variability [43]. Predicted nutrient intakes were adjusted for energy intake using the residual method [44].

### Overall nutrient inadequacy

To assess nutrient adequacy from 24HR data, the individual probability of adequate intake for protein, fiber, calcium, iron, zinc, vitamin A, folate, and vitamin B12 was calculated following the full-probability method for each nutrient, described in the Institute of Medicine guidelines [45], using the energy-adjusted nutrient intakes. Individual overall probability of adequacy was then estimated by tabulating the mean probability of adequacy across those eight nutrients.

The overall nutrient inadequacy outcome was defined as an energy-adjusted mean probability of adequacy across the eight nutrients < 0.5, based on previous validations of the GDQS [8].

### Statistical analysis

Continuous variables were tested for adherence to a normal distribution using the Kolmogorov–Smirnov test. Whenever a continuous variable did not present a normal distribution, non-parametric statistical analysis was applied or the variable was categorized. Categorical variables are presented as relative and absolute frequency while continuous variables are presented as mean (standard error). All descriptive statistics took the complex sample design into consideration (survey mode).

Comparisons of means between sex were conducted using the Mann–Whitney’s test, while comparisons between age ranges and geographical regions were conducted using the Kruskall-Wallis test with Tukey HSD correction for multiple tests.

Spearman’s coefficient was used to assess correlation between GDQS and nutrient intake and for the correlation between MDD-W and nutrient intake. For each nutrient intake, to compare the performance of the GDQS with the MDD-W, Wolfe’s test was applied between the estimated Spearman’s correlation coefficients for each diet metric. Spearman’s coefficient was also used to test the GDQS and MDD-W correlation with UPF intake.

To estimate the odds for nutrient inadequacy across MDD-W and GDQS quintiles, a multiple logistic regression was applied, taking the first quintile as reference, adjusted for age ranges (adolescents, aging from 10 to 19 years; adults, aging from 20 to 59 years; and elderly individuals, aged 60 years or more) urban/rural locality, income (five categories of income), supplement use (yes/no), and recent diet modification (yes/no). The same statistical adjustments were applied to estimate linear trend across quintiles, including the quintile information as a categorical variable in the model, coded “0” for the 1st quintile, “1” for the 2nd quintile, “2” for the 3rd quintile, “3” for the 4th quintiles, and “4” for the 5th quintile. Linear trends in overall nutrient inadequacy across metric quintiles were statistically compared using regression models in which quintiles of GDQS and MDD-W were included in the same model and the parameter estimates associated with 5th quintile were compared using a Wald test (p-difference).

To investigate linear increases in the probability of overall nutrient adequacy across GDQS and MDD-W quintiles, a multiple linear regression model, adjusted for age (years), urban/rural locality, income (five categories of income), supplement use (yes/no), and recent diet modification (yes/no), was used. To check for difference between MDD-W and GDQS performance, Wald’s post-test for first-to-fifth quintile delta difference in the probability of overall nutrient adequacy was applied after the multiple linear regression model.

All tests were repeated, stratifying for sex, age range, and Brazilian major geographic regions to investigate GDQS validity across categories. Except for the application of the National Cancer Institute (NCI) method to adjust for within-person variability, which was carried on using SAS® OnDemand for Academics (SAS Institute Inc., Cary, NC, USA), all statistical analysis were conducted using Stata SE®, version 17.0 (StataCorp LLC, Texas, EUA).

## Results

**Fig. 1:**
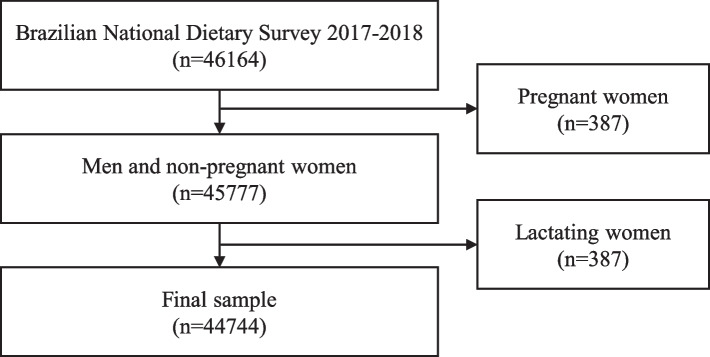
Study flowchart, Brazilian national dietary survey, 2017–2018

After excluding lactating and pregnant women, the final sample included 44,744 individuals aging 10 years and older (Fig. 1). Sex distribution was balanced (51% women and 49% men), and composition was 18% adolescents (10 to 19 years), 64% adults (20 to 59 years), and 18% elderly individuals (> 59 years), which reflects the Brazilian demographic landscape (Table 1). In 2017, most of the Brazilian population lived in urban areas (86%) and, for income distribution, 41% households were earning less than one minimum wage (308 USD in 2017) while only 1% of households had a monthly income above ten minimum wages (3080 USD). In 2017, the Southeast was the most populated region of Brazil (43%), followed by the Northeast (27%), the South (14%), the North, and Midwest (both with 8%).

**Table 1:** Population characteristics, Brazilian individuals aging 10 years or older, Brazilian national dietary survey, 2017–2018

Characteristics	Mean (SE)/%
**Gender**	
Male	49
Female	51
**Age (years)**	43.0 (0.2)
10 – 19	18
20 – 29	16
30 – 39	17
40 – 49	16
50 – 59	15
60 – 69	10
> 70	8
**Self-declared skin color**	
Brown	45
White	43
Black	11
Yellow	0.6
Indigenous	0.4
**Per capita income (USD)**	542 (11)
< 1 MW	41
1 – 2 MW	34
2 – 3 MW	12
3 – 5 MW	7
5 – 10 MW	4
> 10 MW	1
**Household locality**	
Urban	86
Rural	14
**Geographical region**	
North	8
Northeast	27
Southeast	43
South	14
Midwest	8

Mean (SE) and n(%) estimated accounting for complex sample design

*USD* United States dollar, *MW* Minimum wage (308 USD)

**Fig. 2:**
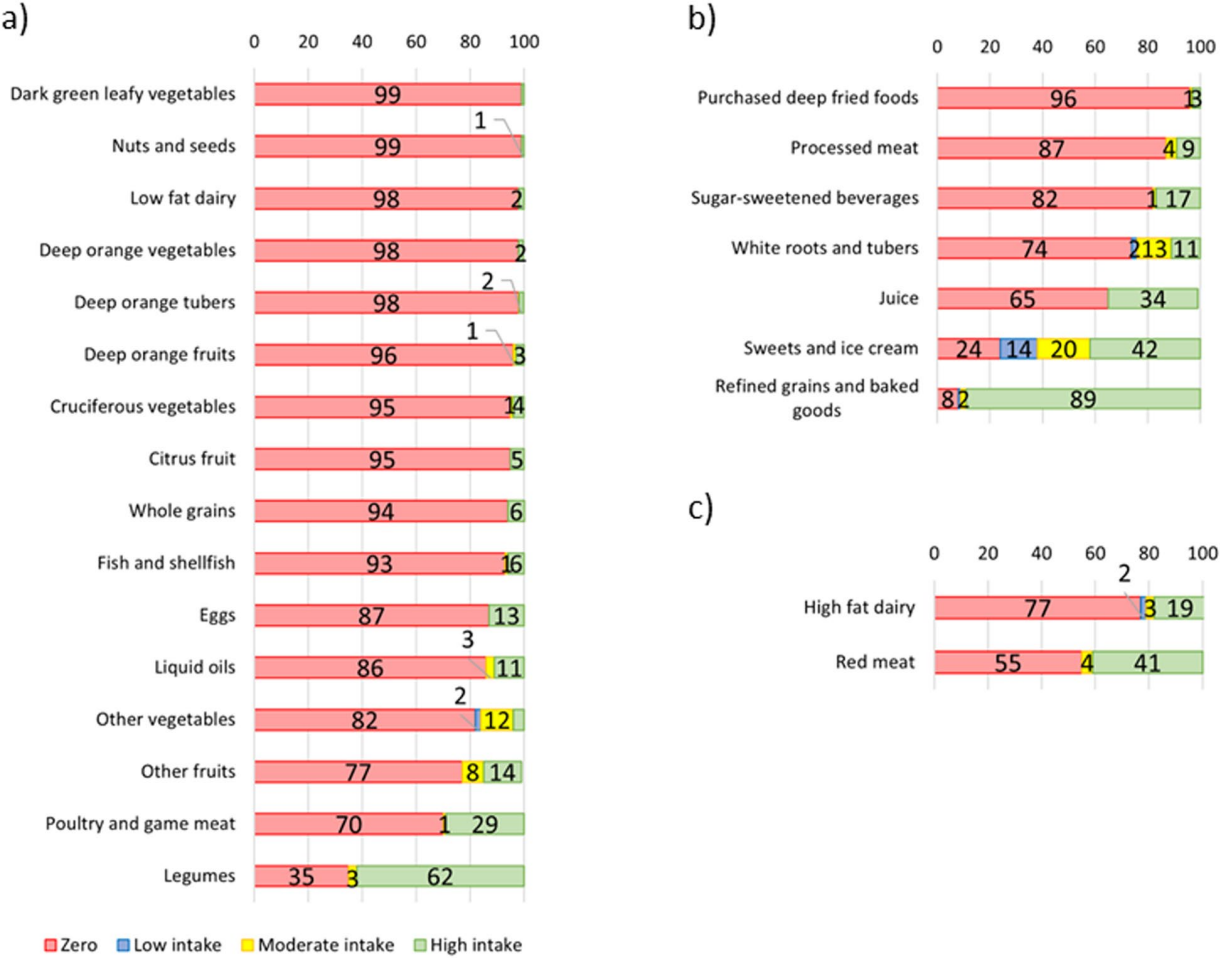
Brazilian diet according to the GDQS components, Brazilian national dietary survey, 2017–2018. Prevalence of zero intake (red), low intake (blue), moderate intake (yellow), and high intake (green) of GDQS healthy (**a**), unhealthy (**b**) and unhealthy in excessive amounts (**c**) food groups intake in the Brazilian population according to GDQS cut-off points. Prevalence was estimated accounting for complex sample design

The mean GDQS for Brazilians was 14.5 (SE = 0.04, out of a 0 to 49 range score), and only 1% of the population had a low-risk diet, while 47% and 52% had high and moderate risk diet, respectively, according to GDQS cut-off points (Table 2). Figure 2 depicts the level of intake for each food component in the Brazilian population and shows that the zero to low intake of fruits and vegetables, nuts and seeds, whole grains, and dairy products (high- and low-fat) along with high intake of refined grains and red meat were the main responsible for the small number of Brazilians with a low-risk diet.

**Table 2:** Dietary characteristics across self-declared sex, Brazilian individuals aging 10 years or older, Brazilian national dietary survey, 2017–2018

Diet characteristic	All Mean (SE)/% *n* = 44,744	Men Mean (SE)/% *n* = 21,460	Women Mean (SE)/% *n* = 23,284	*p* value
**GDQS (score 0 – 49)**	14.50 (0.04)	14.40 (0.04)	14.59 (0.04)	< 0.001
**GDQS categories**				< 0.001
GDQS – high risk (< 15)	52	53	50	
GDQS – moderate risk (15–23)	47	46	48	
GDQS – low risk (≥ 23)	1	1	2	
**MDD-W (score 0 – 10)**	4.83 (0.02)	4.76 (0.02)	4.89 (0.02)	< 0.001
**MDD-W ≥ 5**	54	51	55	< 0.001
**UP (% total energy intake)**	20.9 (0.2)	20.4 (0.3)	21.5 (0.3)	< 0.001
**Mean probability for overall Nutrient adequacy**	58.6 (0.1)	55.9 (0.1)	61.1 (0.1)	< 0.001
**Nutrient adequacy (≥ 50%)**	81.5	77.8	85.2	< 0.001
**Supplement use**				
Any supplement	19	16	22	< 0.001
Vitamins	11	10	12	< 0.001
Minerals	6	3	9	< 0.001
Omega-3	5	3	6	< 0.001
Protein	1.7	2.2	1.3	< 0.001
**Diet modifications**				
Any diet modification	14	10	18	< 0.001
For weight control	5	3	8	< 0.001
For blood pressure control	5	3	6	< 0.001
For cholesterol control	3	2	4	< 0.001
For diabetes treatment	4	3	5	< 0.001
For CVD treatment	0.9	0.8	1.0	0.095

Values are presented as mean (standard error) for continuous variables and relative frequency (%) for categorical variables. Mean (SE) and n (%) estimated accounting for complex sample design. Comparisons of mean values between men and women means were conducted with Mann–Whitney test. Comparison of proportions between men and women were conducted with chi-squared test, in survey mode. *P* values < 0.05 are statistically significant

*CVD* Cardiovascular disease, *GDQS* Global diet quality score, *MDD-W* Minimum dietary diversity for women, *UP* Ultra-processed food

In general, women had better dietary quality than men, with higher GDQS, MDD-W, and probability of overall nutrient adequacy (Table 2). Women, however, presented higher caloric intake from UPF than men (Table 2). Supplement use was more frequent in women than men (one exception observed for protein supplementation, more prevalent in men) (Table 2). The GDQS, MDD-W and probability of nutrient adequacy were progressively higher across age ranges, with adolescents displaying the lowest scores and elderly individuals, the highest (Supplemental Table S 1). Adolescents also had the highest caloric contribution from UPF, 28%, while elderly had the lowest, 17% (Supplemental Table S 1).

Table 3 shows trends in GDQS across household locality and income. The GDQS was higher in households with higher income, a trend also observed for GDQS + and GDQS- (p-trend < 0.001). Lower-income regions of Brazil, North and Northeast, also had lower GDQS compared to the highest-income regions, Southeast and Midwest, for different reasons: in North region, GDQS + was the lowest, pointing to a lower intake of healthy food groups; and in Northeast, GDQS- was the lowest between regions, pointing to a higher intake of unhealthy or unhealthy in excessive amounts food groups.

**Table 3:** GDQS and its sub-metrics means across sociodemographic categories, individuals aging 10 years or older, Brazilian national dietary survey, 2017–2018

**Characteristics**	**GDQS** **Possible range:** **0 to 49** Mean (SE)	**GDQS + ** **Possible range:** **0 to 32** Mean (SE)	**GDQS-** **Possible range:** **0 to 17** Mean (SE)
All	14.50 (0.04)	4.61 (0.03)	9.88 (0.02)
**Household locality**			
Urban	14.51 (0.04)	4.60 (0.04)	9.91 (0.03)
Rural	14.43 (0.07)	4.69 (0.06)	9.74 (0.04)
*P* value	0.393	0.146	0.331
**Brazilian region**			
North	13.55 (0.10)^a^	3.74 (0.09)^a^	9.81 (0.06)^c^
Northeast	14.36 (0.05)^b^	4.84 (0.04)^e^	9.51 (0.03)^a^
Midwest	14.84 (0.09)^d^	4.66 (0.08)^c^	10.18 (0.06)^d^
Southeast	14.80 (0.07)^c^	4.69 (0.07)^d^	10.11 (0.06)^d^
South	14.20 (0.09)^b^	4.42 (0.08)^b^	9.78 (0.05)^b^
*P* value	< 0.001	< 0.001	< 0.001
**Income**			
< 1 MW	14.26 (0.05)	4.44 (0.04)	9.82 (0.03)
1 – 2 MW	14.51 (0.06)	4.66 (0.06)	9.85 (0.04)
2 – 3 MW	14.69 (0.13)	4.72 (0.10)	9.97 (0.08)
3 – 5 MW	14.65 (0.15)	4.79 (0.14)	9.86 (0.12)
5 – 10 MW	15.20 (0.24)	4.94 (0.17)	10.27 (0.12)
> 10 MW	16.77 (0.55)	6.01 (0.48)	10.76 (0.28)
P-trend	< 0.001	< 0.001	< 0.001

*P* values refer to comparison of means non-parametric tests (Mann–Whitney test for comparisons between two groups and Kruskall-Wallis for comparison between more than two groups). Different letters indicate statistically significant differences between groups, after Tukey HSD correction for multiple tests. P-trend refer to the linear increase across income categories and were estimated using a simple linear regression model

*GDQS* Global diet quality score, *MW* Minimum wage

**Fig. 3:**
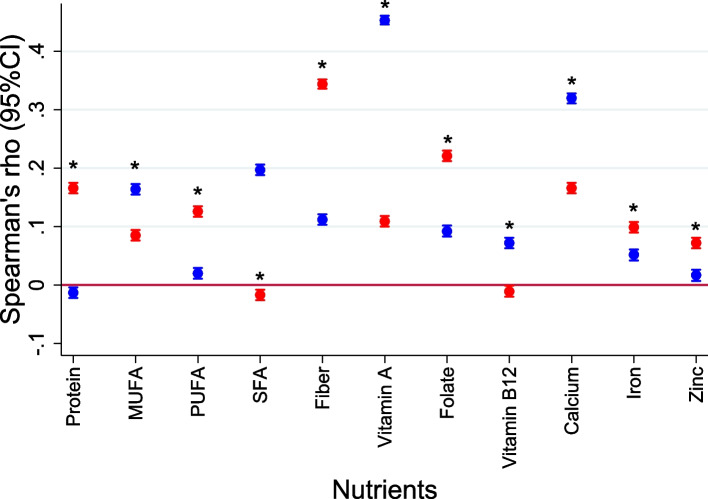
Comparison of Spearman’s correlation coefficient between the Global Diet Quality Score (red) and Minimum Dietary Diversity for Women (blue) with energy-adjusted nutrient intake among Brazilian individuals, Brazilian national dietary survey, 2017. MUFA, monounsaturated fatty acids; PUFA, polyunsaturated fatty acids; SFA, saturated fatty acids. *Best performance metric according to Wolfe’s test

The GDQS had statistically significant correlation with energy-adjusted usual intake for protein, MUFA, PUFA, SFA, dietary fiber, vitamin A, folate, calcium, iron, and zinc intake (Fig. 3, and Supplemental Table S 2). A statistically significant correlation was not observed between the GDQS and energy-adjusted usual intake for vitamin B12 (Fig. 3, and Supplemental Table S 2). The GDQS had better performance than MDD-W for the correlation with protein, PUFA, SFA (for which a negative correlation is expected), dietary fiber, folate, iron, and zinc while MDD-W displayed better performance for MUFA, vitamin A, vitamin B12, and calcium (Fig. 3, and Supplemental Table S 2). Similar results were observed when stratifying for sex, age ranges and geographical regions (Supplemental Tables S 2, S 3, and S 4).

**Fig. 4:**
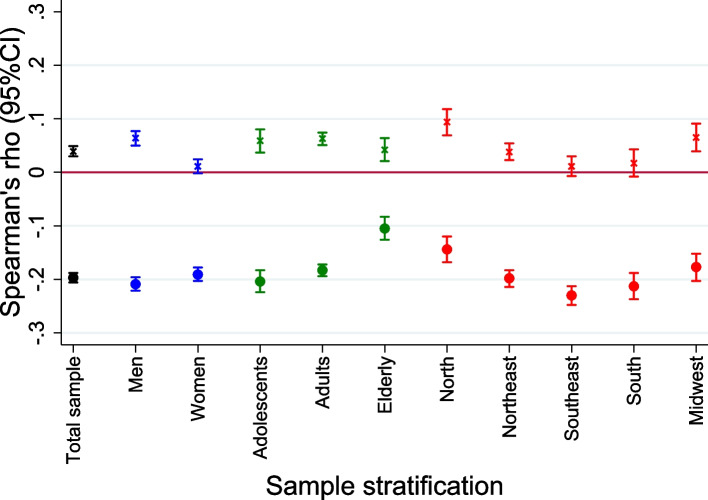
Spearman correlation coefficient (95% Confidence Interval) between the Global Diet Quality Score (circle) and Minimum Diet Diversity for Women (X) with ultra-processed foods caloric contribution in total sample (black) and stratified by sex (blue), age ranges (green), and geographical regions (red), Brazilian National Dietary Survey, 2017–2018

An inverse and statistically significant correlation was found between the GDQS and caloric contribution from UPF intake (rho (95% CI) = -0.20 (-0.21; -0.19)) in the total sample, ranging from -0.23 to -0.11 when stratified by sex, age ranges, and geographical regions), as shown in Fig. 4, while a positive and statistically significant correlation was not found between MDD-W and caloric contribution from UPF intake (rho (95% CI) = 0.04 (0.03; 0.05)) in the total sample, ranging from 0.01 to 0.09 when stratified by sex, age ranges, and geographical regions).

In general, the GDQS + submetric had a weaker correlation with UPF caloric contribution (rho(95%CI) = -0.12 (-0.13; -0.11) in the total sample) than did GDQS- (rho(95%CI) = -0.16 (-0.17; -0.15)) (Supplemental Figure S 2).

**Fig. 5:**
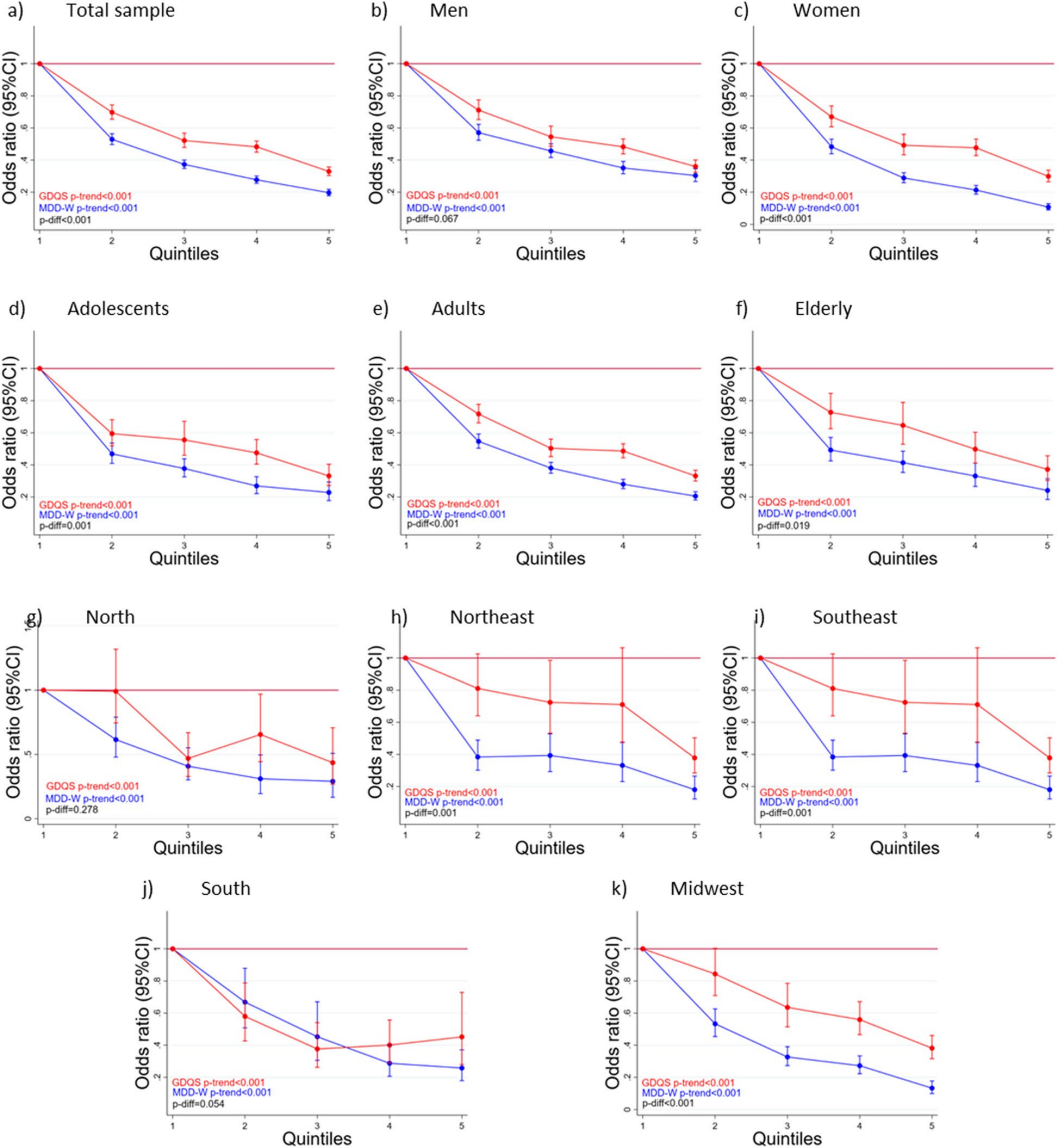
Odds Ratio for nutrient inadequacy across quintiles of the Global Diet Quality Score (red) and the Minimum Dietary Diversity score for Women (blue) in total sample (**a**), and among men (**b**), women (**c**), adolescents (**d**), adults (**e**), and elderly individuals (**f**), and North (**g**), Northeast (**h**), Southeast (**i**), South (**j**), and Midwest (**k**) region residents. Comparison between the two scores was conducted using a multiple logistic model adjusted for age, urban/rural locality, income, supplement use, and recent diet modification, with Wald’s test for difference between the upper quintiles

The odds for nutrient inadequacy (overall nutrient adequacy < 0.5) were lower as quintiles of GDQS and MDD-W were higher, and MDD-W had a slightly but statistically significant better performance in predicting nutrient inadequacy than GDQS, except in men, North, and Midwest region, for which GDQS performance was as good as that observed for MDD-W (p-diff = 0.067, 0.278, and 0.054, respectively) (Fig. 5).

Similarly, Supplemental Figure S 3 shows a linear increase in overall nutrient adequacy across GDQS and MDD-W quintiles. MDD-W had a slightly but statistically significant better performance than GDQS in most stratification categories and total sample, except for men, adolescents, and Midwest region (p-diff = 0.052, 0.056, and 0.121, respectively) (Supplemental Figure S 3).

In general, the GDQS + had similar performance to that of the MDD-W (p-diff = 0.616; Supplemental Figure S 4a) while GDQS- had no association with nutrient inadequacy (p-diff < 0.001; Supplemental Figure S 4b). Similarly, the GDQS + performance for increase in overall nutrient adequacy across quintiles was better than that observed for GDQS- (p-trend =  < 0.001, and 0.019, respectively).

When GDQS risk cut-off points were considered, having a low-risk GDQS (≥ 23) lowered the odds for nutrient inadequacy by 74% (95% CI: 63%—81%) and a moderate-risk GDQS (≥ 15 and < 23) lowered the odds for nutrient inadequacy by 48% (95% CI: 45%—50%). Similar trends were seen when stratifying the sample for sex, and age ranges, as shown in Table 4, with the exception that, in adolescents, the odds in GDQS low-risk category did not reach statistical significance, given the small sample size (*n* = 15). The stratification by geographical regions was not used because North region had zero individuals in the low-risk GDQS category with nutrient inadequacy and, therefore, the analysis could not be conducted.

**Table 4:** Odds ratio for nutrient inadequacy across the Global Diet Quality Score cut-off points, brazilian national dietary survey, 2017–2018

GDQS categories	Total sample (*n* = 44,744)	Men (*n* = 21,460)	Women (*n* = 23,284)	Adolescents (*n* = 8027)	Adults (*n* = 28,604)	Elderly (*n* = 8107)
High risk (score < 15)	1	1	1	1	1	1
Moderate risk	0.52	0.54	0.50	0.55	0.52	0.56
(score 15 – 22)	(0.50 – 0.55)	(0.51 – 0.58)	(0.47 – 0.54)	(0.49 – 0.62)	(0.49 – 0.55)	(0.50 – 0.63)
Low risk	0.26	0.35	0.20	0.80	0.24	0.29
(score ≥ 23)	(0.19 – 0.37)	(0.22 – 0.56)	(0.12 – 0.35)	(0.22 – 2.83)	(0.15 – 0.38)	(0.16 – 0.53)
p-trend	< 0.001	< 0.001	< 0.001	< 0.001	< 0.001	< 0.001

Multiple logistic models adjusted for age, urban/rural locality, income, supplement use, and recent diet modification

## Discussion

The results presented here show, in a nationally representative sample of Brazil, that the GDQS is a good food-based indicator for nutrient intake and overall nutrient adequacy and correlates with the proportion of UPF from diet across sex, age ranges, and geographical stratifications.

The Brazilian diet displayed a low mean GDQS, 14.5 (SE = 0.04, out of a 0 to 49 range score), compared to other population-based studies of Mexican women (mean GDQS = 16.4) [11], Ethiopian women (mean GDQS = 17.4) [16], Chinese women and men (mean GDQS = 19.8) [15], and cohort studies of Indian women (mean GDQS = 23.0) [13], United States women (mean GDQS = 21.6) [20], and ten Sub-Saharan African countries men and women (mean GDQS ranging from 18.0 to 26.0) [18].

A plausible explanation for low mean GDQS in Brazil may be attributed to particularities of the Brazilian dietary habits, such as low intake of fruits and vegetables, nuts and seeds, whole grains, and dairy products (high- and low-fat) along with high intake of refined grains and red meat, and to the fact that the typical Brazilian diet is relatively monotonous. In this sense, Rodrigues and colleagues showed that coffee, white rice, beans, white bread, and red meat were present in 78%, 76%, 60%, 51%, and 38% of all the 24HR collected in HBS 2017–2018, respectively, therefore accounting for most of the daily caloric intake [46].

Fruits and vegetables, on the other hand, besides being consumed by 30% and 45% of the Brazilian population, respectively, are present in insufficient amounts in the Brazilian daily diet, especially in lower-income and food insecure households [46]. In a recent study, Junior and colleagues [47] showed that, fruits and vegetables have high cost per calorie in Brazil and that, to adequate fruits and vegetables intake (> 400 g/day) maintaining food culture and essential nutrient balance, it would be necessary to increase food expenses by around 15% in lower income households (those earning less than one minimum wage), which corresponds to a significant portion of the Brazilian population (41%).

Nuts and seeds, whole grains, and low-fat dairy products, on its turn, are rarely consumed, in part because these foods are not, with rare exceptions, part of the Brazilian food culture, and because the economic access to those products is difficult [46]. Thus, it is not surprising that we found higher GDQS scores in higher-income settings in Brazil, where there is economic access to a wider variety of healthy food products, along with better educational level and access to healthcare services [48].

In fact, similar to the results presented here, higher scores in higher-income settings were also found in studies conducted with other dietary quality metrics in Brazil, such as the Brazilian Healthy Eating Index [48], an indicator of diet-related NCD-risk, and the Planetary Healthy Diet Index [49], an indicator of diet-related planetary health. Among other factors, improvement in quality food availability, education and healthcare access is needed to increase diet quality in Brazil.

On the correlation of GDQS with energy-adjusted nutrient intake in Brazil, GDQS outperformed the MDD-W for most of the tested nutrients, except for MUFA, vitamin B12, vitamin A and calcium, similar to what was observed in other populations [8]. GDQS correlation with MUFA, vitamin A and calcium was, however, in the expected direction and statistically significant, showing that GDQS is a good indicator of energy-adjusted nutrient intake.

These results are explained by the fact that MDD-W was designed to assess adequacy of nutrient intake exclusively, thus, all its variability is dedicated to track the intake of essential vitamins and minerals from food sources [21]. In contrast, the GDQS variability is shared between food sources of essential nutrients and food groups that offer a balanced macronutrient profile, with higher contents of dietary fiber, MUFA, and PUFA, combined with lower contents of SFA, and added sugar, which, ultimately, lowers the strength of its estimated correlation with single micronutrients, although many sources of foods with a balanced macronutrient profile are good sources of vitamins and minerals [8].

The GDQS performance as an indicator of overall nutrient adequacy was similar to those observed in earlier studies for women from Mexico, China, India, Ethiopia, and other Sub-Saharan African countries [8] MDD-W had a slightly but statistically significant better performance in predicting nutrient inadequacy than GDQS in Brazil and Ethiopia. Similar results were seen when the mean difference in overall nutrient adequacy was tested across GDQS quintiles. Those were not unexpected results, since MDD-W was designed to be a sensitive indicator of nutrient adequate intake in women, especially in low-diversity diet settings such as the one found in Brazil and Ethiopia [8, 16].

The GDQS still is advantageous over MDD-W because it is a single indicator of dietary risk for both sides of malnutrition: not only nutrient insufficient intake, but overnutrition and NCD-risk. In a cross-sectional study with a representative sample of Mexican women, for example, there was a linear inverse association of GDQS with BMI, waist circumference, and plasma total and LDL cholesterol [11]. In Chinese women, those in the highest quintile of GDQS had 21% lower odds of having the metabolic syndrome than those in the first quintile [15]. In two longitudinal studies with US and Mexico women, higher GDQS was associated with lower weight gain and lower waist circumference over time [10, 20] and type 2 diabetes risk [19].

Even though the cross-sectional design of the present study and the lack of NCD outcomes preclude us to draw robust conclusions, some of the results presented here point to a possible applicability of the GDQS as a useful indicator of diet NCD-risk in Brazil; for instance, compared to MDD-W, the GDQS correlates better with the intake of nutrients related to NCD risk and prevention (Table S 2 and Fig. 3): positive correlations were found for dietary fiber (GDQS rho = 0.34; MDD-W rho = 0.11; p-diff < 0.001), and PUFA (GDQS rho = 0.13; MDD-W rho = 0.02; p-diff < 0.001), whilst negative correlation were found for SFA (GDQS rho = -0.02; MDD-W rho = 0.20; p-diff < 0.001). Moreover, negative correlation was found between the GDQS and UPF intake (rho (95%CI) = -0.20 (-0.21; -0.19) and 0.04 (0.03; 0.05) for GDQS and MDD-W, respectively). Likewise, an inverse correlation between the GDQS and UPF intake was also seen in a population-based sample of Ethiopian women (rho (95%CI) = -0,27 (-0.34; -0.20)) [17].

Defined as “formulations of ingredients, mostly of exclusive industrial use, typically created by series of industrial techniques and processes” [40], higher UPF intake has been associated with NCD and obesity risk not only in Brazil, but in other countries, such as, USA, France, United Kingdom, Spain, Finland, the Netherlands, Sweden, Japan, China, and South Korea [40, 41, 50]. Moreover, UPF intake has been shown to be a relevant risk factor for overall mortality [41]. The GDQS correlation with UPF intake, then, not only favors the hypothesis that the GDQS is a good indicator of diet NCD-risk, but also shows its alignment with the Brazilian Food Guide, which is based on levels of food processing and sustainable food systems [42].

The present work has some important strengths. Firstly, it is a population-based nationally representative study of the largest country in Latin America, which adds to the worldwide validation of the GDQS and allows comparisons between different geographical regions, food cultures, and socioeconomic strata. Second, the Brazilian Institute of Geography and Statistics has a long history with household surveys in Brazil, with high quality data collection and robust public sharing [30].

Some aspects of the present work, however, must be acknowledged. First, the correlation coefficient between diet metrics (GDQS and MDD-W) and nutrient intake, in spite of being statistically significant, represent moderate-to-low collinearity (ranging from -0.02 to 0.45 in total sample). However, considering the nature of the present study dietary assessment and previous studies conducting similar analysis [8], those were satisfactory results. Moreover, the dietary data collection was conducted using 24HR, which has advantages and disadvantages. While capturing food and food preparation descriptions with more details than other tools which allows flexibility of analysis, as any other tool based on reports, 24HR relies on individuals’ memory, which may be biased. To minimize memory bias, the present study applied the 24HR interview following a structured and automated face-to-face protocol by rigorously trained agents. Furthermore, one single measurement of 24HR does not capture day-by-day variability in individual’s food intake, which can cause underestimation of the intake of foods that are not consumed in a daily basis in Brazil. Hence, the prevalence of zero consumption of many GDQS food groups is inflated, as presented in Fig. 2 (e.g., cruciferous vegetables, fish and seafood, and orange tubers). In accordance, studies conducted in Mexico and Ethiopia, which calculated the GDQS from 24HR and FFQ in different samples of the same population found higher scores from the FFQ data [11, 16]. Thus, future studies planning to conduct primary dietary data collection should consider standardizing the dietary data collection tool to allow comparison across countries and over time. In this sense, a smartphone app has been developed to support this endeavor and automatically score the GDQS in diverse settings [9] and a detailed explanation on how to apply the GDQS in secondary datasets, collected using 24HR or FFQ, can be found elsewhere [8, 51].

Moreover, overall nutrient adequacy, despite being an essential endpoint to validate GDQS as an indicator of adequate micronutrient intake, is not sufficient alone to validate a metric of diet quality. Evaluating NCD-risk endpoints is essential to validate a metric of overall diet quality, and this is better achieved through longitudinal studies. Thus, future research must investigate the relation of GDQS with biomarkers of nutritional status and disease incidence in Brazil to strengthen the evidence supporting the use of GDQS in diet quality surveillance.

It is worth mentioning that many healthy diet metrics have been developed for a diversity of purposes but only a few of those tools were tested for external validity [7, 52]. To our knowledge, among the healthy diet metrics that exist so far, only the GDQS was developed to capture the double burden of malnutrition and had external validity conducted across different nations.

In conclusion, the GDQS is a valid food-based diet metric to assess dietary quality in Brazil, especially regarding nutrient intake, overall probability of nutrient adequate intake, and UPF intake. Future studies must consider longitudinal data analysis and the GDQS association with blood biomarkers in Brazil and worldwide to further investigate the relation between the GDQS and the double burden of malnutrition endpoints, strengthening the recommendation to use this kind of metric to surveillance diet-risks and track progresses towards ending all sorts of malnutrition (UN-SDG number 2) globally.

### Supplementary Information

**Supplementary Material 1.**

## Data Availability

Data sharing is not applicable to this article as no datasets were generated. All datasets were publicly available at the Brazilian Institute of Geography and Statistics online platform and can be downloaded at https://www.ibge.gov.br/estatisticas/sociais/saude/24786-pesquisa-de-orcamentos-familiares-2.html?=&t=downloads (accessed on 23 December 2023).

## Abbreviations

24HR: 24-Hour recalls
AHEI: Alternative Healthy Eating Index
BNDS: Brazilian National Dietary Survey
GDQS: Global Diet Quality Score
HBS: Household Budget Survey
MDD-W: Minimum Diet Diversity for Women
MUFA: Monounsaturated fatty acids
NCD: Non-communicable diseases
PUFA: Polyunsaturated fatty acids
SFA: Saturated fatty acids
UN-SDG: United Nations Sustainable Development Goals
UPF: Ultra-processed foods
