# Supplementary material for: The global diet quality score as an indicator of adequate nutrient intake and dietary quality – a nation-wide representative study
Source: Nutr J. 2024 Apr 17;23:42. doi: 10.1186/s12937-024-00949-x (PMC11022474; doi:10.1186/s12937-024-00949-x)
Supplement: Supplementary file 1 — Supplementary Material 1. [file 12937_2024_949_MOESM1_ESM.docx]

**Supplemental material for:**

**The Global Diet Quality Score as an indicator of adequate nutrient intake and dietary quality – a nation-wide representative study**

Content:

[**Figure S1.** Brazilian geographical regions 2](#_Toc124171444)

[**Table S1**. Dietary characteristics across age ranges, Brazilian individuals aging 10 years or older, Brazilian National Dietary Survey, 2017-2018. 2](#_Toc124171445)

[**Table S2**. Comparison of Spearman correlation between the GDQS and MDD-W with energy-adjusted nutrient intake among Brazilian individuals aging 10 years or older, total sample and stratified by self-declared sex, Brazilian National Dietary Survey, 2017-2018. 3](#_Toc124171446)

[**Table S3**. Comparison of Spearman correlation between the GDQS and MDD-W with energy-adjusted nutrient intake among Brazilian individuals aging 10 years or older, stratified by age ranges, Brazilian National Dietary Survey, 2017-2018. 4](#_Toc124171447)

[**Table S4**. Comparison of Spearman correlation between the GDQS and MDD-W with energy-adjusted nutrient intake among Brazilian individuals aging 10 years or older, stratified by Brazilian geopolitical regions, Brazilian National Dietary Survey, 2017-2018. 5](#_Toc124171448)

[**Figure S2.** Spearman correlation coefficient (95% Confidence Interval) between the GDQS+ (a,b) and GDQS- (c,d) submetrics with ultraprocessed foods intake in percentage of caloric intake (a,c) and in grams (b,d) in total sample (black) and stratified by sex (blue), age ranges (green), and geographical regions (red), Brazilian National Dietary Survey, 2017-2018. 6](#_Toc124171449)

[**Figure S3.** Mean (95% confidence interval) overall nutrient adequacy across quintiles of the Global Diet Quality Score (red) and the Minimum Diet Diversity score for Women (blue) in total sample (a), and among men (b), women (c), adolescents (d), adults (e), and elderly individuals (f), and North (g), Northeast (h), Southeast (i), South (j), and Midwest (k) region residents.. 7](#_Toc124171450)

[**Figure S4**. Odds Ratio for nutrient inadequacy across quintiles of the GDQS + (a) and GDQS – (b) submetrics (red) and the Minimum Diet Diversity score for Women (blue) in total sample, Brazilian National Dietary Survey, 2017-2018.. 8](#_Toc124171451)

[**Figure S5**. Mean (95% confidence interval) overall nutrient adequacy across quintiles of the GDQS + (a) and GDQS – (b) submetrics (red) and the Minimum Diet Diversity score for Women (blue) in total sample, Brazilian National Dietary Survey, 2017-2018. 8](#_Toc124171452)


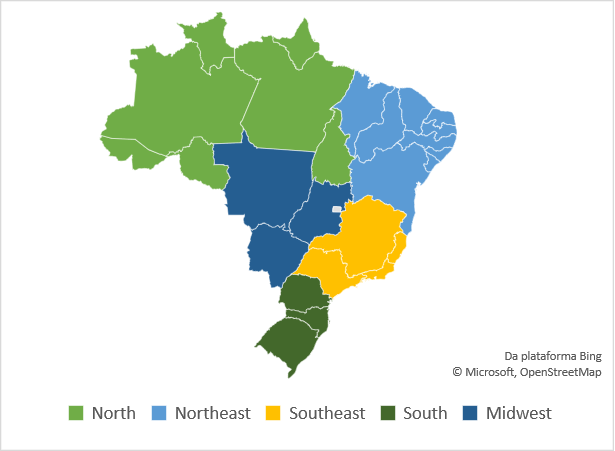


# **Figure S1.** Brazilian geographical regions

# **Table S1**. Dietary characteristics across age ranges, Brazilian individuals aging 10 years or older, Brazilian National Dietary Survey, 2017-2018.

| Diet characteristic | Adolescents,  10 to 19 y  Mean (SE)/%  n=8027 | Adults,  20 to 59 y  Mean (SE)/%  n=28604 | Elderly,  > 60 y  Mean (SE)/%  n=8107 | P value |
| --- | --- | --- | --- | --- |
| **GDQS (score 0 – 46)** | 13.64 (0.04)^a^ | 14.47 (0.02)^b^ | 15.43 (0.04)^c^ | <0.001 |
| **GDQS categories** |  |  |  | <0.001 |
| GDQS – high risk (<15) | 62 | 52 | 42 |  |
| GDQS – moderate risk (15 -23) | 38 | 46 | 56 |  |
| GDQS – low risk (≥23) | 0 | 1 | 3 |  |
| **MDD-W (score 0 – 10)** | 4.52 (0.04)^a^ | 4.86 (0.02)^b^ | 5.02 (0.04)^c^ | <0.001 |
| **MDD-W >5** | 48 | 57 | 62 | <0.001 |
| **UP (% total energy intake)** | 27.8 (0.3)^a^ | 20.2 (0.3)^b^ | 16.7 (0.3)^c^ | <0.001 |
| **Energy-adjusted overall nutrient adequacy (%)** | 56.9 (0.5)^a^ | 58.1 (0.3)^b^ | 62.0 (0.5)^c^ | <0.001 |
| **Nutrient adequacy (>50%)** | 80 | 81 | 85 | <0.001 |
| **Supplement use** |  |  |  |  |
| Any supplement | 10 | 17 | 34 | <0.001 |
| Vitamins | 7 | 10 | 17 | <0.001 |
| Minerals | 2 | 4 | 16 | <0.001 |
| Omega-3 | 0.7 | 3.9 | 10.7 | <0.001 |
| Protein | 1.3 | 2.2 | 0.4 | <0.001 |
| **Diet modifications** |  |  |  |  |
| Any diet modification | 4 | 13 | 27 | <0.001 |
| For weight control | 3 | 7 | 3 | <0.001 |
| For blood pressure control | 0.1 | 3.3 | 14.6 | <0.001 |
| For cholesterol control | 0.3 | 2.0 | 9.3 | <0.001 |
| For diabetes treatment | 0.3 | 2.2 | 11.8 | <0.001 |
| For CVD treatment | 0 | 0.5 | 2.9 | <0.001 |

CVD – cardiovascular disease ; GDQS – Global Diet Quality Score; MDD-W – Minimum Diet Diversity for Women; UP – ultraprocessed food. Values are presented as mean (standard error) for continuous variables and relative frequency (%) for categorical variables. Comparison between men and women means was conducted with Mann-Whitney test and between men and women proportions was conducted with Pearson chi-squared test, in survey mode. P values <0.05 are statistically significant.

# **Table S2**. Comparison of Spearman correlation between the GDQS and MDD-W with energy-adjusted nutrient intake among Brazilian individuals aging 10 years or older, total sample and stratified by self-declared sex, Brazilian National Dietary Survey, 2017-2018.

| Sample | Nutrient | GDQS rho | MDD-W rho | Wolfe’s test |
| --- | --- | --- | --- | --- |
| All | Protein | 0.17* | -0.01* | <0.001 |
| (n=44744) | MUFA | 0.09* | 0.16* | <0.001 |
|  | PUFA | 0.13* | 0.02* | <0.001 |
|  | SFA | -0.02* | 0.20* | <0.001 |
|  | Fiber | 0.34* | 0.11* | <0.001 |
|  | Vitamin A | 0.11* | 0.45* | <0.001 |
|  | Folate | 0.22* | 0.09* | <0.001 |
|  | Vitamin B12 | -0.01 | 0.07* | <0.001 |
|  | Calcium | 0.17* | 0.32* | <0.001 |
|  | Iron | 0.10* | 0.05* | <0.001 |
|  | Zinc | 0.07* | 0.02* | <0.001 |
| Males | Protein | 0.18* | -0.03* | <0.001 |
| (n=21460) | MUFA | 0.09* | 0.14* | <0.001 |
|  | PUFA | 0.15* | 0.02* | <0.001 |
|  | SFA | -0.02* | 0.18* | <0.001 |
|  | Fiber | 0.33* | 0.09* | <0.001 |
|  | Vitamin A | 0.08* | 0.45* | <0.001 |
|  | Folate | 0.22* | 0.09* | <0.001 |
|  | Vitamin B12 | -0.01 | 0.06* | <0.001 |
|  | Calcium | 0.14* | 0.30* | <0.001 |
|  | Iron | 0.11* | 0.06* | <0.001 |
|  | Zinc | 0.08* | <\|0.01\|* | <0.001 |
| Females | Protein | 0.16* | 0.01* | <0.001 |
| (n=23284) | MUFA | 0.08* | 0.18* | <0.001 |
|  | PUFA | 0.11* | 0.03* | <0.001 |
|  | SFA | -0.02* | 0.21* | <0.001 |
|  | Fiber | 0.37* | 0.15* | <0.001 |
|  | Vitamin A | 0.13* | 0.45* | <0.001 |
|  | Folate | 0.23* | 0.11* | <0.001 |
|  | Vitamin B12 | -0.01 | 0.09* | <0.001 |
|  | Calcium | 0.19* | 0.34* | <0.001 |
|  | Iron | 0.09* | 0.06* | <0.001 |
|  | Zinc | 0.07* | 0.03* | <0.001 |

GDQS – Global Diet Quality Score; MDD-W – Minimum Diet Diversity for Women; MUFA – monounsaturated fatty acids; PUFA – polyunsaturated fatty acids; SFA – saturated fatty acids. * Statistically significant Spearman’s correlation coefficient (p<0.05). Wolfe-s test for difference between Spearman correlation coefficients from GDQS and MDD-W. The metric with the best performance was colored in green.

# **Table S3**. Comparison of Spearman correlation between the GDQS and MDD-W with energy-adjusted nutrient intake among Brazilian individuals aging 10 years or older, stratified by age ranges, Brazilian National Dietary Survey, 2017-2018.

| Age range | Nutrient | GDQS rho | MDD-W rho | Wolfe’s test |
| --- | --- | --- | --- | --- |
| Adolescents | Protein | 0.20* | <\|0.01\| | <0.001 |
| 10 to 19 y.o. | MUFA | 0.03* | 0.11* | <0.001 |
| (n=8027) | PUFA | 0.13* | <\|0.01\| | <0.001 |
|  | SFA | -0.05* | 0.16* | <0.001 |
|  | Fiber | 0.37* | 0.12* | <0.001 |
|  | Vitamin A | 0.08* | 0.40* | <0.001 |
|  | Folate | 0.26* | 0.11* | <0.001 |
|  | Vitamin B12 | 0.01 | 0.07* | <0.001 |
|  | Calcium | 0.14* | 0.29* | <0.001 |
|  | Iron | 0.16* | 0.10* | <0.001 |
|  | Zinc | 0.11* | 0.04* | <0.001 |
| Adults | Protein | 0.16* | -0.02* | <0.001 |
| 20 to 59 y.o. | MUFA | 0.10* | 0.18* | <0.001 |
| (n=28604) | PUFA | 0.14* | 0.02* | <0.001 |
|  | SFA | -0.02* | 0.21* | <0.001 |
|  | Fiber | 0.33* | 0.09* | <0.001 |
|  | Vitamin A | 0.09* | 0.46* | <0.001 |
|  | Folate | 0.22* | 0.08* | <0.001 |
|  | Vitamin B12 | -0.02 | 0.07* | <0.001 |
|  | Calcium | 0.15* | 0.32* | <0.001 |
|  | Iron | 0.10* | 0.04* | <0.001 |
|  | Zinc | 0.07* | 0.01* | <0.001 |
| Elderly | Protein | 0.13* | -0.02* | <0.001 |
| > 60 y.o. | MUFA | 0.11* | 0.18* | <0.001 |
| (n=8107) | PUFA | 0.12* | 0.05* | <0.001 |
|  | SFA | 0.02 | 0.21* | <0.001 |
|  | Fiber | 0.33* | 0.14* | <0.001 |
|  | Vitamin A | 0.13* | 0.46* | <0.001 |
|  | Folate | 0.22* | 0.12* | <0.001 |
|  | Vitamin B12 | <\|0.01\| | 0.07* | <0.001 |
|  | Calcium | 0.22* | 0.34* | <0.001 |
|  | Iron | 0.08* | 0.05* | 0.020 |
|  | Zinc | 0.04* | <\|0.01\| | 0.002 |

GDQS – Global Diet Quality Score; MDD-W – Minimum Diet Diversity for Women; MUFA – monounsaturated fatty acids; PUFA – polyunsaturated fatty acids; SFA – saturated fatty acids. * Statistically significant Spearman’s correlation coefficient (p<0.05). Wolfe-s test for difference between Spearman correlation coefficients from GDQS and MDD-W. The metric with the best performance was colored in green.

# **Table S4**. Comparison of Spearman correlation between the GDQS and MDD-W with energy-adjusted nutrient intake among Brazilian individuals aging 10 years or older, stratified by Brazilian geopolitical regions, Brazilian National Dietary Survey, 2017-2018.

| Region | Nutrient | GDQS rho | MDD-W rho | Wolfe’s test |
| --- | --- | --- | --- | --- |
| North | Protein | 0.18* | -0.04 | <0.001 |
| (n=3641) | MUFA | 0.06* | 0.14* | <0.001 |
|  | PUFA | 0.16* | 0.05* | <0.001 |
|  | SFA | -0.02 | 0.17* | <0.001 |
|  | Fiber | 0.40* | 0.25* | <0.001 |
|  | Vitamin A | 0.05* | 0.38* | <0.001 |
|  | Folate | 0.26* | 0.20* | <0.001 |
|  | Vitamin B12 | 0.02 | <\|0.01\| | 0.033 |
|  | Calcium | 0.18* | 0.22* | 0.024 |
|  | Iron | 0.12* | 0.15* | 0.0198 |
|  | Zinc | 0.11* | 0.10* | 0.248 |
| Northeast | Protein | 0.18* | -0.03* | <0.001 |
| (n=12017) | MUFA | 0.03* | 0.08* | <0.001 |
|  | PUFA | 0.11* | -0.08* | <0.001 |
|  | SFA | -0.05* | 0.20* | <0.001 |
|  | Fiber | 0.30* | 0.07* | <0.001 |
|  | Vitamin A | 0.05* | 0.46* | <0.001 |
|  | Folate | 0.19* | 0.06* | <0.001 |
|  | Vitamin B12 | -0.01 | 0.11* | <0.001 |
|  | Calcium | 0.15* | 0.33* | <0.001 |
|  | Iron | 0.06* | 0.04* | 0.046 |
|  | Zinc | 0.07* | -0.03* | <0.001 |
| Southeast | Protein | 0.19* | 0.06* | <0.001 |
| (n=19163) | MUFA | 0.13* | 0.22* | <0.001 |
|  | PUFA | 0.09* | 0.04* | <0.001 |
|  | SFA | <\|0.01\| | 0.20* | <0.001 |
|  | Fiber | 0.30* | 0.05* | <0.001 |
|  | Vitamin A | 0.15* | 0.48* | <0.001 |
|  | Folate | 0.19* | <\|0.01\| | <0.001 |
|  | Vitamin B12 | 0.02* | 0.14* | <0.001 |
|  | Calcium | 0.16* | 0.33* | <0.001 |
|  | Iron | 0.09* | -0.03 | <0.001 |
|  | Zinc | 0.08* | 0.05* | 0.002 |
| South | Protein | 0.12* | 0.03 | <0.001 |
| (n=6491) | MUFA | 0.13* | 0.19* | 0.012 |
|  | PUFA | 0.12* | 0.03* | <0.001 |
|  | SFA | -0.03* | 0.15* | <0.001 |
|  | Fiber | 0.41* | 0.15* | <0.001 |
|  | Vitamin A | 0.18* | 0.41* | <0.001 |
|  | Folate | 0.20* | 0.08* | <0.001 |
|  | Vitamin B12 | <\|0.01\| | 0.13* | <0.001 |
|  | Calcium | 0.17* | 0.30* | <0.001 |
|  | Iron | 0.08* | 0.02 | <0.001 |
|  | Zinc | 0.07* | 0.01 | <0.001 |
| Midwest | Protein | 0.18* | -0.03* | <0.001 |
| (n=3432) | MUFA | 0.11* | 0.15* | 0.026 |
|  | PUFA | 0.13* | 0.02 | <0.001 |
|  | SFA | 0.02 | 0.16* | <0.001 |
|  | Fiber | 0.29* | 0.06* | <0.001 |
|  | Vitamin A | 0.13* | 0.47* | <0.001 |
|  | Folate | 0.20* | 0.05* | <0.001 |
|  | Vitamin B12 | 0.04* | 0.03* | 0.686 |
|  | Calcium | 0.20* | 0.30* | <0.001 |
|  | Iron | 0.11* | 0.01 | <0.001 |
|  | Zinc | 0.05* | <\|0.01\| | <0.001 |

GDQS – Global Diet Quality Score; MDD-W – Minimum Diet Diversity for Women; MUFA – monounsaturated fatty acids; PUFA – polyunsaturated fatty acids; SFA – saturated fatty acids. * Statistically significant Spearman’s correlation coefficient (p<0.05). Wolfe-s test for difference between Spearman correlation coefficients from GDQS and MDD-W. The metric with the best performance was colored in green.

| a) | b) |
| --- | --- |
| **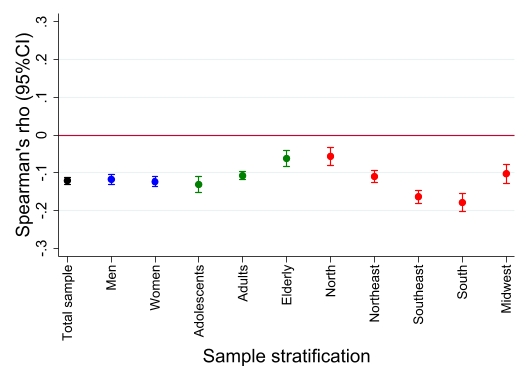** | **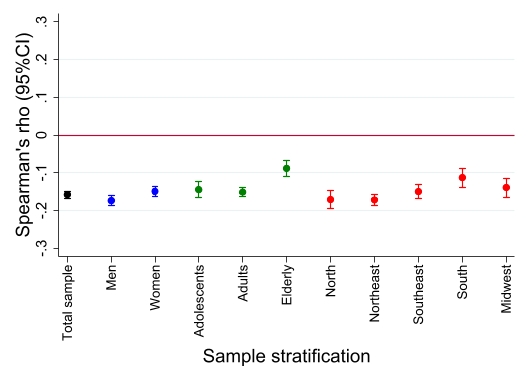** |

# **Figure S2.** Spearman correlation coefficient (95% Confidence Interval) between the GDQS+ (a,b) and GDQS- (c,d) submetrics with ultraprocessed foods intake in percentage of caloric intake (a,c) and in grams (b,d) in total sample (black) and stratified by sex (blue), age ranges (green), and geographical regions (red), Brazilian National Dietary Survey, 2017-2018.

| 1. Total sample |  | 1. Men | | |  | 1. Women |
| --- | --- | --- | --- | --- | --- | --- |
| 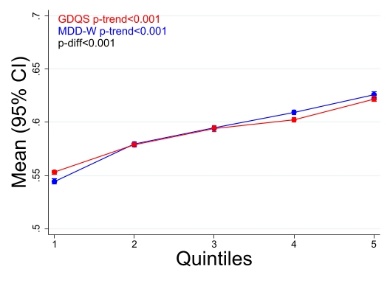 |  | 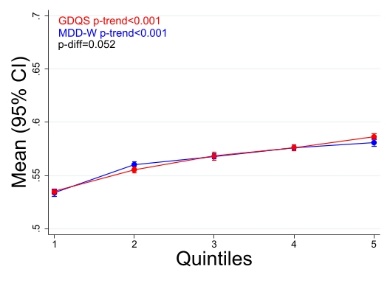 | | |  | 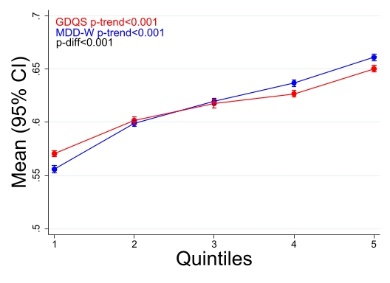 |
| 1. Adolescents |  | 1. Adults | | |  | 1. Elderly |
| 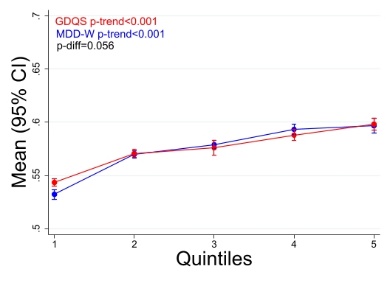 |  | 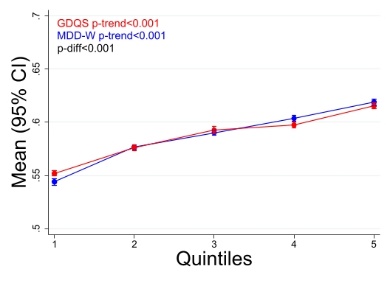 | | |  | 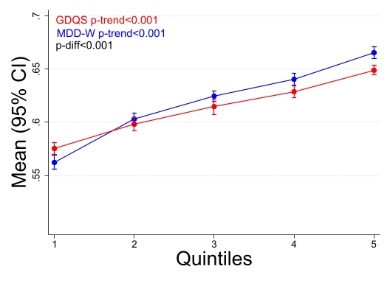 |
| 1. North |  | 1. Northeast | | |  | 1. Southeast |
| 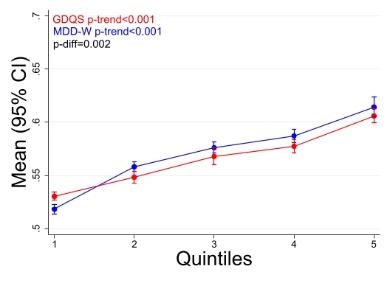 |  | 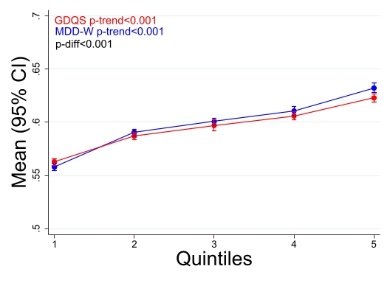 | | |  | 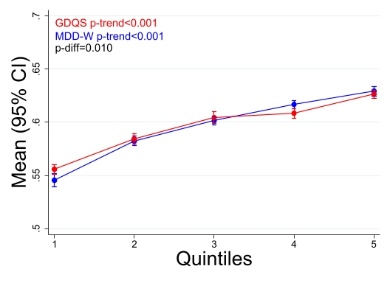 |
| 1. South | | |  | 1. Midwest | | |
| 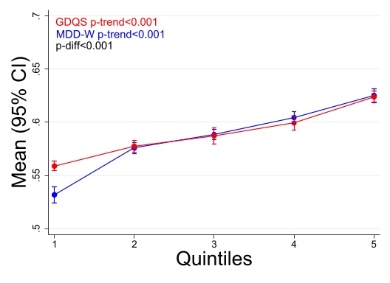 | | |  | 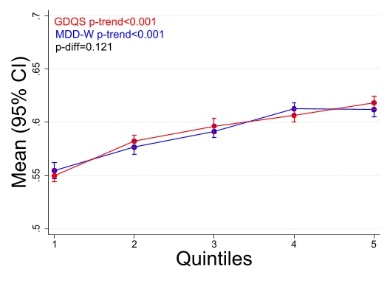 | | |

# **Figure S3.** Mean (95% confidence interval) overall nutrient adequacy across quintiles of the Global Diet Quality Score (red) and the Minimum Diet Diversity score for Women (blue) in total sample (a), and among men (b), women (c), adolescents (d), adults (e), and elderly individuals (f), and North (g), Northeast (h), Southeast (i), South (j), and Midwest (k) region residents. Multiple linear model adjusted for age, urban/rural locality, income, supplement use, and recent diet modification, with Wald’s test for difference between the upper quintiles.

| 1. **GDQS +** |  | 1. **GDQS -** |
| --- | --- | --- |
| **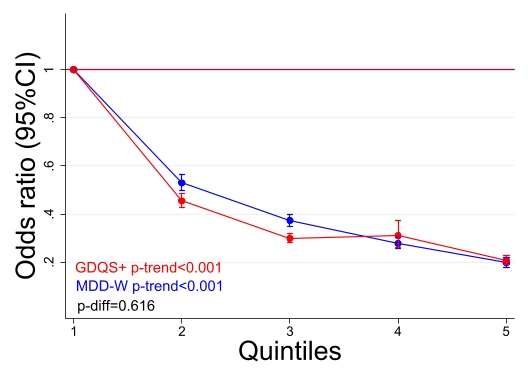** |  | **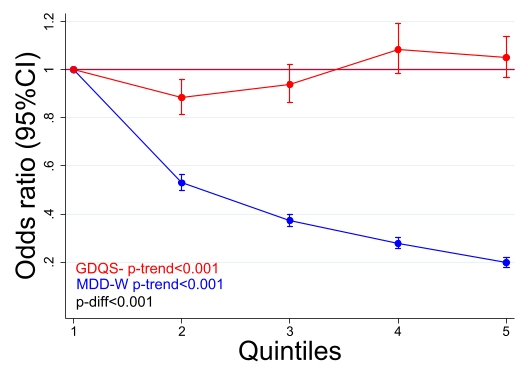** |

# **Figure S4**. Odds Ratio for nutrient inadequacy across quintiles of the GDQS + (a) and GDQS – (b) submetrics (red) and the Minimum Diet Diversity score for Women (blue) in total sample, Brazilian National Dietary Survey, 2017-2018. Multiple logistic model adjusted for age, urban/rural locality, income, supplement use, and recent diet modification, with Wald’s test for difference between the upper quintiles.

| 1. **GDQS +** |  | 1. **GDQS -** |
| --- | --- | --- |
| **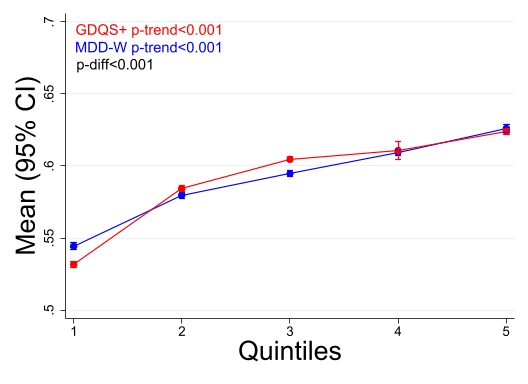** |  | **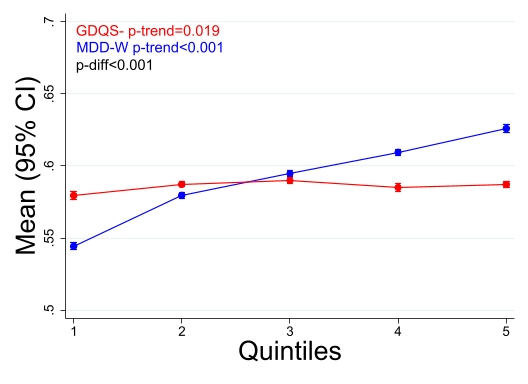** |

# **Figure S5**. Mean (95% confidence interval) overall nutrient adequacy across quintiles of the GDQS + (a) and GDQS – (b) submetrics (red) and the Minimum Diet Diversity score for Women (blue) in total sample, Brazilian National Dietary Survey, 2017-2018. Multiple linear model adjusted for age, urban/rural locality, income, supplement use, and recent diet modification, with Wald’s test for difference between the upper quintiles
